# Improving the Expression of Human Granulocyte Colony Stimulating Factor in Escherichia coli by Reducing the GC-content and Increasing mRNA Folding Free Energy at 5’-Terminal End

**DOI:** 10.34172/apb.2020.073

**Published:** 2020-08-09

**Authors:** Kartika Sari Dewi, Asrul Muhamad Fuad

**Affiliations:** Research Center for Biotechnology, Indonesian Institute of Sciences, Cibinong, Bogor, Indonesia, 16911.

**Keywords:** GCSF, mRNA folding free energy, GC-content, 5'-terminal end, Synonymous codon substitution, Codon optimization

## Abstract

***Purpose:*** Strategy for improving the production of biopharmaceutical protein continues to develop due to increasing market demand. Human granulocyte colony stimulating factor (hG-CSF) is one of biopharmaceutical proteins that has many applications, and easily produced in *Escherichia coli* expression system. Previous studies reported that codon usage, rare codon, mRNA folding and GC-content at 5’-terminal end were crucial for protein production in *E. coli*. In the present study, the effect of reducing the GC-content and increasing the mRNA folding free energy at the 5’-terminal end on the expression level of hG-CSF proteins was investigated.

***Methods:*** Synonymous codon substitutions were performed to generate mutant variants of open reading frame (ORF) with lower GC-content at 5’-terminal ends. Oligoanalyzer tool was used to calculate the GC content of eight codons sequence after ATG. Whereas, mRNA folding free energy was predicted using KineFold and RNAfold tools. The template DNA was amplified using three variant forward primers and one same reverse primer. Those DNA fragments were individually cloned into pJexpress414 expression vector and were confirmed using restriction and DNA sequencing analyses. The confirmed constructs were transformed into *E. coli* NiCo21(DE3) host cells and the recombinant protein was expressed using IPTG-induction. Total protein obtained were characterized using SDS-PAGE, Western blot and ImageJ software analyses.

***Results:*** The result showed that the mutant variant with lower GC-content and higher mRNA folding free energy near the translation initiation region (TIR) could produce a higher amount of hG-CSF proteins compared to the original gene sequence.

***Conclusion:*** This study emphasized the important role of the nucleotide composition immediately downstream the start codon to achieve high-yield protein product on heterologous expression in *E. coli*.

## Introduction

Granulocyte-colony stimulating factor (G-CSF), also known as colony-stimulating factor 3 (CSF3), is a glycoprotein that acts in haematopoiesis by controlling the production, survival, proliferation, differentiation and function of granulocytes that comprises 70% of white blood cells.^1-3^ Recombinant human G-CSF has many pharmaceutical applications. In oncology, it has been used especially for the prevention of chemotherapy-induced neutropenia and also mobilisation of peripheral blood progenitor cells in a healthy donor before collection in hematopoietic stem cell transplantation.^4,5^

Human G-CSF is a 19 kDa single-chain polypeptide consisting of 174 amino acids which contains five Cysteines forming two intramolecular disulfide bridges between Cys36-Cys42 and Cys64-Cys74, and one free Cysteine at residue 17.^6-8^ Naturally occurred G-CSF has an O-linked glycosylation at Thr133 which protects the proteins from aggregation without affecting its biological activity.^9^

As a host organism for producing recombinant proteins, *E. coli* has many advantages including simple nutrient requirement, high growth rate on low cost substrate, well characterized on molecular genetics and cellular physiology.^8^ It also classified as safe organism for producing recombinant protein products.^10^ The non-glycosylated G-CSF produced in *E. coli* can be biologically active as produced in mammalian cell, but differs in the addition of N-terminal methionine.^11,12^

As one of most important biopharmaceuticals worldwide, recombinant G-CSF production should be optimized in order to get a high-yield protein product with low production cost. In addition to the choice of expression system and other environmental conditions, several crucial factors at the genetic level should be considered such as codon usage, rare codons composition, mRNA secondary structure and GC-content near the translation initiation region (TIR).^13^

Many studies reported that nucleotide composition at 5’-terminal end of the open reading frame (ORF) or near the TIR is often a bottleneck in recombinant protein production. Theoretically, strong folding at 5’-terminal ends can cover the area of ribosome binding site and inhibits the translation initiation rate. Reducing the GC-content in this region is known can improve the protein expression level in *E.* coli.^14-16^ Whereas, higher value of mRNA folding free energy indicates more unstable hairpin, which is preferred in expressing recombinant proteins.

In the present study, effects of reducing the GC content and increasing mRNA folding free energy at 5’-terminal end on G-CSF protein expression level were investigated. Two mutant variants of ORF were made by reducing the GC-content of eight codons immediately downstream the start codon from 75% to 62.5% and 54.2% which performed through synonymous codons substitutions. The expression of G-CSF was regulated under T7 promoter in *E. coli* expression system and monitored using SDS PAGE and western blot analyses.

## Materials and Methods

### Microbial strains and plasmid

The *E. coli* strain TOP10 was used for cloning and *E. coli* NiCo21(DE3) was used for protein expression. Plasmid pJexpress414 (ATUM, Newark, California, United States) was used as the expression vector.

### Media

Lysogeny Broth (LB) medium [1% (w/v) Tryptone, 0.5% (w/v) Yeast Extract, and 1% (w/v) NaCl] was used for both cloning and expression step. Except for making *E. coli* competent cells and negative control, LB medium supplemented with 100 µg/mL ampicillin (LB-amp) was used in this whole experiment.

### Primer design and template

PET21-hGCSF, which already constructed by Fuad et al,^17^ was used as the template for PCR amplification. Three forward primers were designed in this experiment (Table 1). One of them was designed based on the original hG-CSF template and not modified, while the others two were 5’-ends terminally modified and designed for synonymous codon substitution. However, the GC content reduction at 5’-ends were calculated using Oligoanalyzer 3.1 and minimum free energy (MFE) of local mRNA secondary structure formation near the rbs were monitored using KineFold and RNAfold web server.^18-20^ To facilitate the cloning step, all forward primers were added with *Nde* I restriction site, whereas the reverse primer was added with *Xho* I restriction site. All primers were purchased from Integrated DNA Technologies (IDT, Singapore).

**Table 1:** The Design of Forward and Reverse Primers

**Name of Primer**	**Description**	**Sequence of Primer**	**GC Content (%)**	**Tm (°C)**
hG-CSF1	Not modified	5’TATACATATGACCCCGCTTGGCCCTGCGAGC 3’	58.1	67.3
hG-CSF2	5’-ends terminally modified	5’TATACATATGACACCACTTGGCCCAGCAAGCTCCCTGC 3’	52.6	67.8
hG-CSF3	5’-ends terminally modified	5’TATACATATGACACCACTTGGCCCTGCATCATCTCTGCCCC 3’	51.2	67.8
R	Reverse primer	5’ATGACTCGAGTCATTACGGCTGCG CCAGATGACGC 3’	57.1	58.6

### PCR and cloning

Three variants of the ORF encoding the same recombinant hG-CSF protein were amplified from pET21-hGCSF. PCR amplification was carried out separately for each pair of primers and conducted as follows: initial denaturation at 95°C for 5 minutes; 30 cycles of denaturation at 95°C for 1 minute, annealing at 66.8°C for 30 seconds, and extension at 72°C for 1 minute, then final extension at 72°C for 5 minutes. PCR product was purified using a PCR purification kit (Qiagen, Hilden, Germany). Purified PCR product and pJExpress414 expression vector (ATUM, Newark, California, United States) were exposed to *Nde* I/*Xho* I double digestion (3 IU/µg) (Thermo Fisher Scientific, Waltham, Massachusetts, United States). Digested products were examined with agarose gel electrophoresis and isolated from agarose gel using Gel/PCR DNA Fragments Extraction Kit (Geneaid, New Taipei City, Taiwan). Each ORF was cloned into an expression vector using T4-DNA ligase enzyme (2 IU/50 ng plasmid) (Kapa Biosystems, Inc., Wilmington, Massachusetts, United States) and carried out for 72 hours at 4°C. The plasmids obtained were named pJ414-hGCSF1, pJ414-hGCSF2 and pJ414-hGCSF3.

### Transformation and characterization

The ligation product was transformed into *E. coli* TOP10 using one-step transformation and stock solution (TSS) by Chung et al,^21^ then continued with heat-shock method as described by Froger and Hall with minor modification.^22^ The transformants were selected in LB-amp medium at room temperature for 20 hours. Recombinant plasmids from *E. coli* TOP10 transformants were isolated using alkaline lysis method according to Green and Sambrook^23^ and characterized by restriction analyses, subsequently sequenced to ensure that the synonymous substitution at 5’- terminal ends was successfully performed (1^st^ BASE, Selangor, Malaysia). The amino acid sequence of hG-CSF1, hG-CSF2, and hG-CSF3 were deduced using Translate Tools (ExPaSy) continued with alignment analysis using Pairwise Sequence Alignment Tools (EMBL-EBI).^24,25^ Confirmed recombinant plasmids were then transformed into *E. coli* NiCo21(DE3) and then characterized by colony PCR. The colony PCR was done using 2 µL of culture as a template and PCR protocol as mentioned above.

### Growth condition for expression of hG-CSF

Each variant of *E. coli* NiCo21(DE3) transformants was grown overnight in 1 mL LB-amp medium at 25°C, 150 rpm. This culture was diluted 1:50 with LB-amp medium and grown at 25°C, 150 rpm until 0D_600_~1 was reached. After that, IPTG with a final concentration of 1 mM was added and growth was continued overnight at 25°C, 150 rpm. *E. coli* NiCo21(DE3) non-transformant was used as a negative control which had the same treatment as above.

### Protein characterization

The sample for total proteins analyses was made by adding 50 µL of sample buffer and 50 µL of denaturing lysis buffer (8M urea, 10 mM Tris-Cl, and 100 mM NaH_2_PO_4_) into pellet cells from 1 mL culture, then boiled for 15 m. About 1 µL of sample was subjected to SDS-polyacrylamide gel electrophoresis (SDS-PAGE) according to Laemmli on 15% polyacrylamide gel.^26^

Western blot analysis was carried out using a method as described in Dewi et al.^27^ In this experiment, anti-G-CSF mouse monoclonal IgG antibody (1:2000 dilution, Santa Cruz Biotechnology Inc., Santa Cruz, California, United States) was used as primary antibody, while AP-conjugated goat anti-mouse IgG antibody (1:3500 dilution, Santa Cruz Biotechnology, Inc., Santa Cruz, California, United States) was used as secondary antibody. Both SDS-PAGE and Western blot analyses used 1.5 µg filgrastim (Neupogen®, Kirin-Amgen Inc., Thousand Oaks, California, United States) as a standard for recombinant h-G-CSF proteins. Finally, the amount of recombinant hG-CSF proteins which expressed from pJ414-hGCSF1, pJ414-hGCSF2, and pJ414-hGCSF3 recombinant plasmids were calculated using ImageJ software.^28^

## Results and Discussion

### Synonymous codon substitution

To observe the effects of GC content on local mRNA secondary structure formation and hG-CSF expression level, a synonymous substitution was performed to alter several codon sequences immediately downstream the start codon. Codon usage table of *Escherichia coli* B from Kazusa Codon Database (http://www.kazusa.or.jp) was used to select the appropriate codons for synonymous substitution.^29^ In this study, two mutant variants of hG-CSF1 ORF has been constructed. About eight codons encoding T P L G P A S S amino acids were undergone GC content reduction which calculated using Oligoanalyzer 3.1 web server.^18^ The codon adaptation index (CAI) of eight codon sequences was calculated using CAIcal web server.^30^ After those analyses have been conducted, the forward primers were made for PCR amplification.

Table 2 shows that the GC content and CAI value of eight codons were decreased sequentially from hG-CSF1, hG-CSF2 and hG-CSF3. The MFE required for mRNA secondary structure formation around the area of ribosome binding site (rbs) was predicted using RNAfold and KineFold web server.^19,20^ Table 3 shows that a higher GC content has a lower folding free energy. The higher value of MFE is considered the more unstable mRNA secondary structure which preferred for protein expression.^31^

**Table 2:** GC content and codon adaptation index (CAI) of 8 codons immediately downstream the start codon

**ORF**	**Nucleotide sequences**	**GC content**	**CAI**
hG-CSF1	ACC CCG CTT GGC CCT GCG AGC AGC	75%	0.695
hG-CSF2	ACA CCA CTT GGC CCA GCA AGC TCC	62.5%	0.462
hG-CSF3	ACA CCA CTT GGC CCT GCA TCA TCT	54.2%	0.418

**Table 3:** Minimum free energy for mRNA secondary structure formation around the 5’-terminal end

**ORF**	**Nucleotide sequences**	**Minimum Free Energy (Kcal/mol)**	
		**RNAfold**	**Kinefold**
hG-CSF1	CC TCT AGA AAT AAT TTT GTT TAAC TTT T**AG GAG GT**A AAA CAT ATG ACC CCG CTT GGC CCT GCG AGC AGC CTG CCC	-12.4	-15.9
hG-CSF-2	CC TCT AGA AAT AAT TTT GTT TAAC TTT T**AG GAG GT**A AAA CAT ATG ACA CCA CTT GGC CCA GCA AGC TCC CTG CCC	-8.9	-12.4
hG-CSF-3	CC TCT AGA AAT AAT TTT GTT TAAC TTT T**AG GAG GT**A AAA CAT ATG ACA CCA CTT GGC CCT GCA TCA TCT CTG CCC	-7.3	-10.8

Note: The sequences in blue color are ribosome binding site.

**Figure 1:**
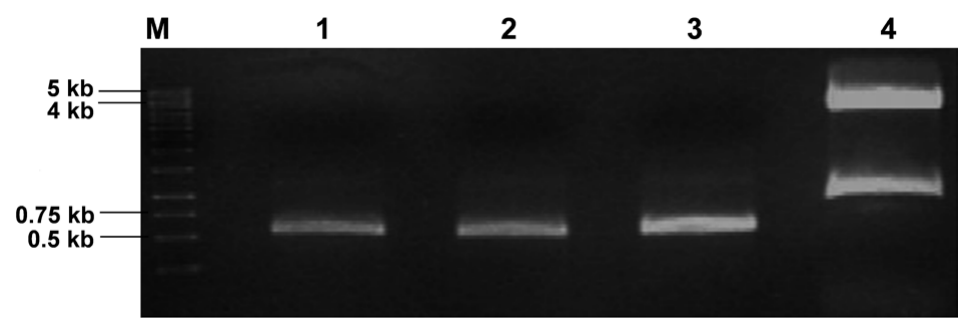
Preparation of expression vector and insert DNA by *Nde* I/*Xho* I double digestion. Lane 1-3, ORF of hG-CSF1, hG-CSF2 and hG-CSF3, respectively; Lane 4, recombinant pJExpression414 expression vector with previous insert DNA.

Plasmid construction was performed to obtain recombinant plasmid carrying ORF encoding hG-CSF proteins. All variants of hG-CSF ORF were amplified from pET21-hGCSF and the result showed 0.53 kb of PCR product (Figure not shown). The expression vector and insert DNA were prepared by double digestion of pJExpress414 and PCR products using *Nde* I and *Xho* I restriction enzymes (Figure 1). Lane 4 showed result of pJExpress414 digestion which consisting plasmid backbone (4.3 kb) and previous insert (0.75 kb).

To ensure a good balance between stability of annealed DNA ends and activity of DNA ligase, thus ligation of cohesive DNA ends is normally carried out at 12-16°C for 24 hours.^32^ However, a lower temperature has advantage in decreasing the random motion in ligation mix, enable the cohesive ends to find their partner. The hydrogen bonds were subsequently formed and remained by the time DNA ligase helps phosphodiester bonds formation. Because a low temperature will reduce DNA ligase activity, thus a longer incubation time was used in this experiment.

**Figure 2:**
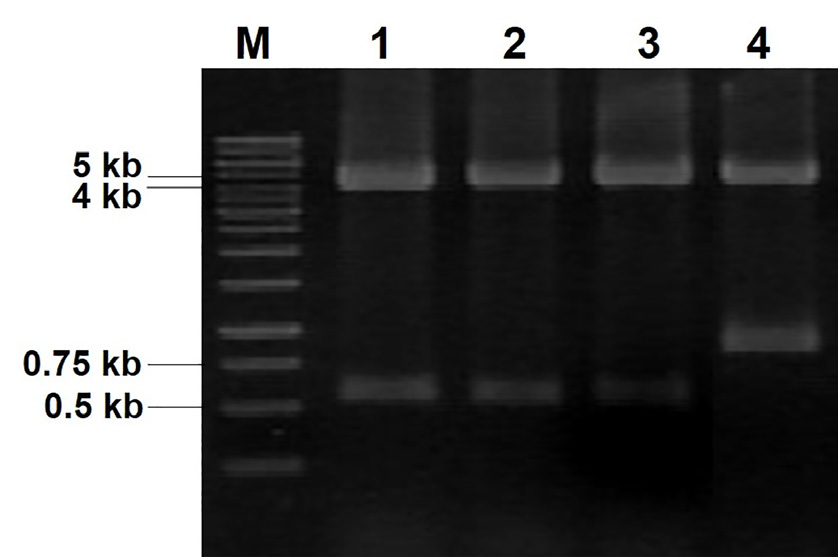
Restriction analysis of recombinant plasmids by *Nde* I/*Xho* I double digestion. Lane 1-3, pJ414-hGCSF1, pJ414-hGCSF2, pJ414-hGCSF3, respectively; Lane 4, recombinant pJExpress414 expression vector with previous insert DNA.

The recombinant plasmids were isolated from growing *E. coli* TOP10 transformants and characterized by restriction and DNA sequencing analyses. Restriction analysis was performed to determine the actual size of the expression vector and insert DNA. Figure 2 shows double digestion of recombinant plasmids using *Nde* I and *Xho* I restriction enzymes, resulting 2 DNA bands with size of 4.3 kb and 0.53 kb which corresponds to the theoretical size of pJExpress414 expression vector and hG-CSF ORF, respectively. PJExpress414 with previous insert DNA was included in this analysis as a negative control.

**Figure 3:**
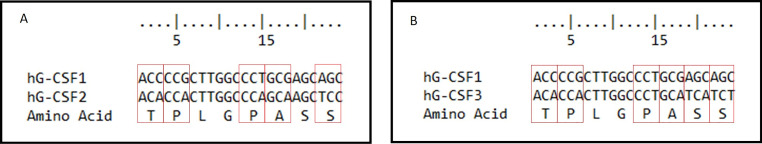
Pairwise sequences alignment between (A) hG-CSF 1 and hG-CSF2; (B) hG-CSF1 and hG-CSF3. The synonymous mutation was marked within a box.

The DNA sequencing analysis and amino acids sequences alignment were carried out to ensure that synonymous substitution was successfully achieved. Figure 3A and 3B shows the nucleotide sequences of some initial codons which already been substituted. Those DNA sequences were translated into amino acid and then aligned to one and another to ensure that no amino acids have been changed. The result of these analyses concluded that the three variants of recombinant plasmids were successfully constructed without altering the amino acids composition.

**Figure 4:**
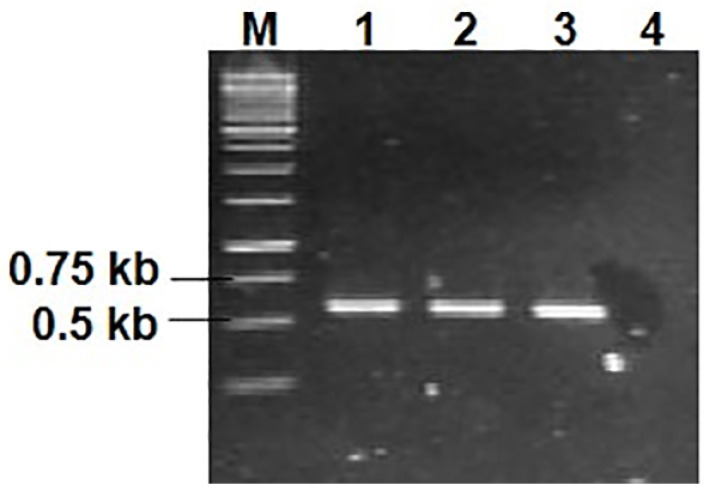
Colony PCR analysis of *E. coli* NiCo21(DE3) transformants using specific primers. Lane 1-3, amplification fragment from colony carrying pJ414-hGCSF1, pJ414-hGCSF2, and pJ414-hGCSF3, respectively; lane 4, negative control.

All the confirmed recombinant plasmid were then transformed into *E. coli* NiCo21(DE3) and characterized by colony PCR using specific primers. This screening method was important because not all colonies appear following transformation carrying plasmid with the desired DNA fragment.^33^ Figure 4 shows the colony PCR from colonies carrying pJ414-hGCSF1, pJ414-hGCSF2, and pJ414-hGCSF3 expression vector. The positive results were indicated by the presence of DNA bands with size of 0.53 kb (Figure 4).

### Expression and characterization of hG-CSF proteins

**Figure 5:**
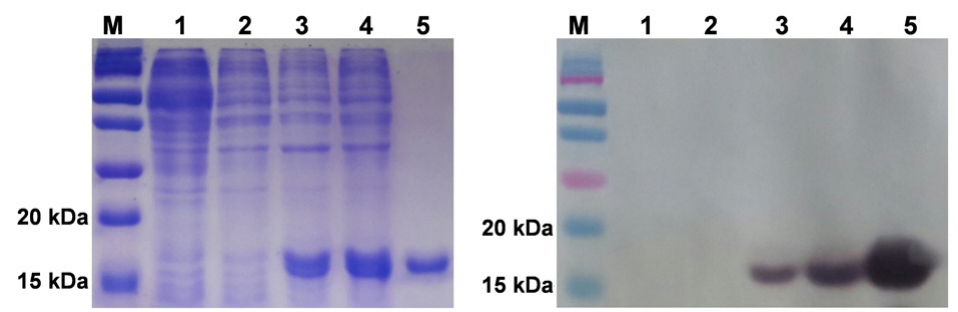
(A) SDS-PAGE and (B) Western blot analyses of hG-CSF proteins. Lane 1, total proteins of untransformed *E. coli* NiCo21(DE3); Lane 2-4, total proteins of hG-CSF1, hG-CSF2 and hG-CSF3, respectively; Lane 5, filgrastim as a standard protein.

To determine the expression level of recombinant hG-CSF proteins which already been genetically modified, the positive colonies were grown in LBamp and induced with IPTG. Neupogen as a standard was included in SDS-PAGE and Western Blot analyses. The hG-CSF band is deduced to have the approximate size of 18.8 kDa as it is shown on the electrophoregram (Figure 5A). However, there was no band with a size of 18.8 kDa detected in hG-CSF1 as it found in hG-CSF2 and hG-CSF3. It is indicated that expression of hG-CSF1 proteins did not occur or very weak expression. Therefore, Western blot analysis was carried out to prove that the prediction from SDS-PAGE analysis was correct.

Compared to SDS-PAGE analysis, Western Blot has better sensitivity and specificity to protein detection. This technique has the ability to detect picogram levels of protein in a sample. Besides, the specific interaction between antibody and antigen allows the detection of target protein in complex mixtures containing >100,000 different proteins.^34^ However, the result of western blot analysis showed that no protein band with size of 18.8 kDa was detected in hG-CSF1 (Figure 5B), even though we used anti-hG-CSF monoclonal antibody which has a high specificity. This result proved that the hG-CSF1 proteins cannot be expressed in *E. coli* NiCo21(DE3) expression system. ImageJ software was used to calculate the total proteins of hG-CSF2 and hG-CSF3 in overproduction culture. The results of this analysis showed that the yield of hG-CSF2 and hG-CSF3 were 31.44 and 73.4 µg/mL culture, respectively.

In this study, we compared the heterologous expression of three variants of ORF encoding hG-CSF in *E. coli* NiCo21(DE3). This research was conducted based on many reports of previous studies which stated that there was a correlation between nucleotides composition at 5’-terminal ends and protein expression level.^14-16,35^ In addition, it has been known that the distribution of codon usage frequency at the 5’-terminal ends was different to that observed in the rest of the ORF.^36-39^ The obtained results provide important information in choosing strategies to increase protein expression level of hG-CSF through genetic improvement.

Kudla et al reported that there is a strong correlation between mRNA folding and protein synthesis.^14^ Their study suggested that tightly folded mRNA near the start codon will inhibit translation initiation rate. In this case, high GC content at the 5’-terminal ends were correlated with low mRNA folding free energy which increases the stability of mRNA secondary structure formation. It makes thermodynamic sense. GC pair has three hydrogen bonds while AU pair has only two, thus GC-rich mRNA tends to fold into more stable structure than AU-rich. Hence, synonymous substitutions around the start codon can regulate protein expression.

Their findings lead to the following prediction: Adding a stretch of codons with weak folded mRNA which indicated with high mRNA folding free energy at the 5’-terminal ends should increase protein expression, even if the additional codons have low CAI.^14^ Our results were consistent with their findings. We have found that lowering GC content of DNA encoding hG-CSF immediately downstream the start codon could increase mRNA folding free energy, and resulted in more unstable mRNA secondary structure which leads to increase protein expression level.

Our findings were also in line with research by Gu et al^16^ which suggested that decreased mRNA stability at the translation-initiation region facilitates efficient start-codon recognition.^16^ The ability of mRNA to sequester ribosomes will be inhibited when local mRNA secondary structure is present, thus lowering the effectiveness of translation initiation rate.^13^ Previously, Jia et al^40^ were also stated that the stability of mRNA secondary structure is an important factor which influences protein expression levels.^40^ Theoretically, if Shine-Dalgarno sequence and start codon itself tends to be base-paired to other nucleotides, then they will not interact efficiently with the pre-initiation complex/30S subunits.^13^

Moreover, we have found that the optimization of only 8 codons immediately downstream the start codon was enough in order to improve the expression level of recombinant proteins in *E. coli* expression system. This result was better than the previous study by Ghavim et al which stated that optimization of 20 first codons was enough to achieve high expression level of recombinant proteins in *E. coli.*^41^

According to our results, the highest protein expression level was achieved when GC content at 5’-terminal ends reduced from 75% to 54.2% along with increasing the MFE from -12.4 Kcal/mol to -7.3 kcal/mol using RNAfold web server and -15.9 kcal/mol to -10.8 kcal/mol using Kinefold web server. These results prove that GC content reduction and unstable mRNA secondary structure at 5’-terminal ends resulted in mRNA efficiency for translation.

## Conclusion

In brief, our study proves that lowering the GC-content at 5’-terminal ends, exactly on eight codons downstream the ATG, has a great impact to improve the expression level of recombinant hG-CSF proteins in *E. coli* . Based on our findings, trials of GC-content reduction through synonymous codon substitutions near the start codon is recommended for improving the expression level of recombinant proteins in *E. coli* expression system, while preventing problems associated with the mRNA secondary structure formation.

## Ethical Issues

Not applicable.

## Conflict of Interest

There is no conflict of interest.

## Acknowledgments

This research was supported by Indonesian Institute of Sciences Flagship Program during 2015-2017 fiscal years.
